# Air-Coupled Ultrasonic Receivers with High Electromechanical Coupling PMN-32%PT Strip-Like Piezoelectric Elements

**DOI:** 10.3390/s17102365

**Published:** 2017-10-16

**Authors:** Rymantas J. Kazys, Reimondas Sliteris, Justina Sestoke

**Affiliations:** Ultrasound Institute of Kaunas University of Technology, LT-51423 Kaunas, Lithuania; rymantas.kazys@ktu.lt (R.J.K.); reimondas.sliteris@ktu.lt (R.S.)

**Keywords:** air-coupled ultrasonic transducers, PMN-32%PT crystals, ultrasonic array

## Abstract

For improvement of the efficiency of air-coupled ultrasonic transducers PMN-32%PT piezoelectric crystals which possess very high piezoelectric properties may be used. The electromechanical coupling factor of such crystals for all main vibration modes such as the thickness extension and transverse extension modes is more than 0.9. Operation of ultrasonic transducers with such piezoelectric elements in transmitting and receiving modes is rather different. Therefore, for transmission and reception of ultrasonic signals, separate piezoelectric elements with different dimensions must be used. The objective of this research was development of novel air-coupled ultrasonic receivers with PMN-32%PT strip-like piezoelectric elements vibrating in a transverse-extension mode with electromechanically controlled operation and suitable for applications in ultrasonic arrays. Performance of piezoelectric receivers made of the PMN-32%PT strip-like elements vibrating in this mode may be efficiently controlled by selecting geometry of the electrodes covering side surfaces of the piezoelectric element. It is equivalent to introduction of electromechanical damping which does not require any additional backing element. For this purpose; we have proposed the continuous electrodes to divide into two pairs of electrodes. The one pair is used to pick up the electric signal; another one is exploited for electromechanical damping. Two types of electrodes may be used—rectangular or non-rectangular—with a gap between them directed at some angle, usually 45°. The frequency bandwidth is wider (up to 9 kHz) in the case of non-rectangular electrodes. The strip-like acoustic matching element bonded to the tip of the PMN-32%PT crystal may significantly enhance the performance of the ultrasonic receiver. It was proposed to use for this purpose AIREX T10.110 rigid polymer foam, the acoustic impedance of which is close to the optimal value necessary for matching with air. It was found that in order to get a wide bandwidth the length of the matching strip should be selected not a quarter wavelength λ/4 at the antiresonance frequency but at lower frequency. It allowed achieving the frequency bandwidth (14–18)% with respect to the central frequency at −3 dB level.

## 1. Introduction

Air-coupled ultrasonics is a non-contact technique which is already used in non-destructive testing, material characterization [1,2,3,4,5] and even for secure wireless transmission of data [6,7]. Such a technique has many advantages over conventional ultrasonic techniques. The primary advantage of air-coupled ultrasonics is that there is no contact between the object under investigation and the ultrasonic transducer. For this reason, it is ideally suited for inspecting materials susceptible to interaction with or damage by water, including wood [8,9], some foams, certain composites [10], paper [11] and even foodstuffs [12,13]. Ultrasonic air-coupled methods are already being used for the characterization of elastic properties of various materials, especially in the case when direct acoustic contact with the investigated material is not allowed [1,8]. Applications of non-contact inspection range from testing of automotive tires [14,15] to the inspections in the aircraft structures [16,17,18]. Modern airliners use significant amounts of composite materials reducing their weight [19,20]. Composites have found increasing application in commercial aircraft structures because of the strength, stiffness, fatigue and corrosion resistance and weight benefits jointly contributing to the improvement of performance [21]. Airborne ultrasound is a useful non-contact method of detecting and imaging defects in aging aircraft structures, given efficient transducers for use as transmitters and receivers are available [16,18].

A spatial resolution of ultrasonic NDT techniques is improving with an increased frequency, which may reach even a few MHz [4]. However, during the last years the air-coupled ultrasonics was applied and for testing of civil structures such as concrete structures. Due to attenuation and scattering of ultrasonic waves in concrete, usually frequencies lower than 100 kHz are used. Progress in this field is impeded by a lack of efficient air-coupled transducers in the frequency band up to 50 kHz [2,3]. On the other hand the frequency range used for a secure transmission of data is (50–100) kHz [7]. It means that a frequency range below 100 kHz becomes attractive for various air-coupled applications.

The main currently encountered problem is big losses of ultrasonic signals up to (120–150) dB, which are caused by the attenuation and significant mismatch of acoustic impedances of ultrasonic transducers and air. The acoustic impedance of air is 0.445 kRayl at a room temperature and 30–35 MRayl of most piezoelectric materials. One of the ways of solving this issue is an application of piezoelectric composite whose specific acoustic impedance is lower than that of monolithic piezoelectric ceramics [22,23,24,25]. Further enhancement of the performance of ultrasonic air-coupled transducers is limited by the piezoelectric effect of the piezoelectric material being used. For this purpose, the 1–3 connectivity PMN-32%PT single crystal composites were used and their performance was compared with the sensitivity of the 1–3 PZT ceramic piezocomposites. It was found that sensitivities are very similar but the single crystal composites possessed a wider bandwidth up to 89% with respect to the central frequency, however their operation frequency range is between (0.5–1.5) MHz [26]. Performance of the 1–3 connectivity piezoelectric composite transducers may be improved by matching layers made of materials with low acoustic impedance, for example, polypropylene foam ferroelectric film [27,28,29,30]. 

Another option is application of polyvinylidene fluoride (PVDF) films [31,32,33]. A relatively strong piezoelectric response and mechanical durability make it a valuable material for use in surface acoustic wave (SAW) devices and air-coupled transducers [31]. The piezoelectric polymer PVDF is flexible, has a low density (*ρ* = 1780 kg/m^3^), a low acoustic impedance (*Z* = 3 MRayl) and a high piezoelectric constant (*g*_33_ = 0.14–0.33 Vm/N). However the electromechanic coupling factor *k_t_* is rather low (0.1–0.15) [32] what makes it inefficient as a transmitter of ultrasonic waves. The dielectric constant is also low (*ε* = 10–12) and consequently the electric input or output impedance is quite high (>1 MΩ). Application of such films in low frequency air-coupled arrays is complicated due to their flexibility and high output electric impedance.

Acoustic matching problems may be avoided by application of capacitive ultrasonic transducers [1]. Electrostatic or capacitive transducers consist of a flexible membrane and a back plate or substrate. The membrane is made of a thin metal-plated polymer film, thickness of which is a few microns. The membrane may be also manufactured using microelectronic technologies [34,35]. A constant high voltage and driving electric signal are applied between the membrane and back plate and they force the membrane to vibrate. Such transducer possesses a quite wide bandwidth but usually operate in the frequency range higher than a few hundreds of kHz [34,35,36]. In addition, they are not yet applied in low frequency air-coupled ultrasonic arrays.

For non-destructive testing of composite structures, guided waves are increasingly being used. Such waves may be excited by air-coupled techniques and they are well suited for a fast testing of large areas [17,19,24,37,38,39,40]. Nowadays air-coupled instrumentation enables to achieve a scanning speed of flat components 500 mm/s [20]. It has been shown that guided waves in composite structures for non-destructive evaluation may be efficiently excited and picked-up by air-coupled ultrasonic linear arrays [41]. Such arrays at frequencies lower than 50 kHz enable excitation of anti-symmetric Lamb wave modes with velocities close or even lower than ultrasound velocity in air. It means that in this case there are no losses due to a wave leaking to air, that allows covering longer distances for a non-destructive testing [11,16]. For this purpose, we have proposed air-coupled multi-element array in which as individual elements piezoelectric strips made of PMN-32%PT crystals vibrating in a transverse-extension mode are used [42]. PMN-32%PT crystals possess a very high piezoelectric properties. The electromechanical coupling factor for the transverse extension mode is *k*_32_ > (0.84–0.91) [42,43,44]. The Curie temperature for the PMN-32%PT crystals is 90 °C [44]. It allows using them in majority of NDT applications. Performance of such elements and the array as a whole in a transmitting mode was described in detail in [42]. It was shown that exploitation of a transverse extension mode allows developing efficient air-coupled ultrasonic transmitters in a low frequency range up to 100 kHz. For radiation of ultrasonic waves, the tip of the piezoelectric strip is exploited. The aperture in this case is rather small 1 × 5 mm, e.g., a few times smaller than the wavelength of ultrasonic wave in air at 40 kHz, but such dimensions enable to use such strips in electrically steered ultrasonic arrays. The developed transducer possesses low transduction losses of −11.4 dB at the frequency *f_r_* = 41.5 kHz [42]. However, the performed investigation limited to only a transmission mode. 

In the case of a high electromechanical coupling, such as that observed in PMN-32%PT type single crystal piezoelectric elements novel problems and possibilities arise. Mainly they are caused by a high electromechanical coupling coefficient of the PMN-32%PT strips vibrating in a transverse-extension mode. The arising problems are the following. 

Frequencies of maximal transmission and reception usually coincide with the resonance and antiresonance frequencies of the piezoelectric element respectively. Due to a very high electromechanical coupling coefficient, those frequencies are very different. For example in the case of 20 × 5 × 1 mm^3^ PMN-32%PT strip vibrating in a transverse-extension mode the maximum of a transmission coefficient is obtained at the resonance frequency *f_r_* = 31.4 kHz and maximum of the reception is obtained at the antiresonance frequency *f_a_* = 52.8 kHz, e.g., the difference is even 21.4 kHz. It means that the same element cannot efficiently operate in a transmitting-receiving mode and for that, two separate piezoelectric elements with different dimensions must be used. 

On the other hand, we have found that the frequency bandwidth even in the case of the air-coupled transducers with acoustic matching elements between the piezoelectric element and air is rather narrow-less than 3%, what results in a long transient process in a pulse mode duration of which is longer than 50 periods. Such transducers are not suitable for many NDT techniques. The frequency bandwidth may be widened by a mechanical damping, for example by means of a special backing attached to the back end of a piezoelectric element, but we found that in this case transduction losses increase by −20 dB. 

The named problems may be solved in the following ways:

- For transmission and reception of ultrasonic waves to use separate piezoelectric elements of different dimensions in which a resonance frequency of the transmitter is corresponds to the antiresonance frequency of the receiver.
- In the transverse-extension mode propagation direction of the ultrasonic wave is orthogonal to the direction of the electric field inside the piezoelectric element. It allows manipulating geometry of the electrodes covering side surfaces of the piezoelectric element to control frequency and pulse responses of the transducer. It is equivalent to introduction of an electromechanically controlled damping, which in the case of a high electromechanical coupling coefficient which in our case is *k*_32_ > (0.84–0.91) [42,43,44] may be very strong.

Therefore, objective of this research was development of novel air-coupled ultrasonic receivers with PMN-32%PT strip-like piezoelectric elements vibrating in a transverse-extension mode with electromechanically controlled operation and suitable for applications in ultrasonic arrays.

## 2. Electromechanical Damping of a Strip-Like Receiver

**Figure 1:**
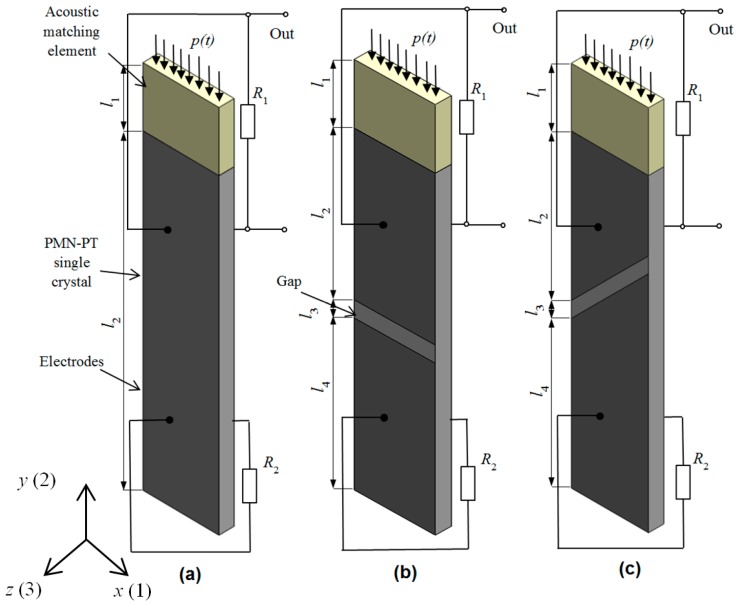
Piezoelectric strip-like receivers operating in a transverse-extension mode: (**a**)—with continuous electrodes; (**b**)—with two rectangular electrodes—case A; (**c**)—with two non-rectangular electrodes—case B; *p(t)* is the incident acoustic pressure.

For reception of ultrasonic waves strip-like PMN-32%PT piezoelectric elements vibrating in a transverse-extension mode may be used. Exploitation of such mode enables to achieve rather low operation frequencies (<150 kHz) what is necessary for many air-coupled applications [2,3,7,8]. PMN-32%PT piezoelectric crystals are anisotropic therefore in order to get a high sensitivity the piezolelectric strips were cut of bigger crystal plates in the crystallographic direction 2. In this case, a very high electromechanical coupling coefficient up to 0.91 is observed [42,43,44]. For reception of an ultrasonic wave the rectangular tip of the strip is exploited (Figure 1a). Of course, the active area in this case is rather small, but a set of such strip-like receivers may be assembled into a multi-element array similar as in the case described by us in [43]. The incident ultrasonic wave excites electric charges on lateral surfaces of the piezoelectric strip, which are coated by electrodes. For improvement of the sensitivity and the frequency bandwidth, the acoustic strip-like matching element attached to the tip of the piezoelectric element similar as in the case of transmitting transducer is used [43]. Lateral dimensions of the matching strip are the same as of the piezoelectric element.

A further improvement of the frequency bandwidth of the transducer may be obtained dividing the lateral continuous electrodes into two pairs of electrodes (Figure 1b,c). Then for the signal reception is exploited one pair (top) of electrodes, the second lower pair acts as electromechanically controlled backing element. The degree of damping may be controlled by changing resistance of the resistor *R*_2_ connected to the bottom pair of electrodes. There may be various geometries of electrodes exploited, for example rectangular (Figure 1b) or non-rectangular (Figure 1c).

First, we have analyzed the case with two rectangular electrodes, e.g., when the gap between electrodes is oriented at 90° angle with respect to the longer edges of the piezoelectric element. The performance of the proposed receivers theoretically was investigated by a finite elements method (FEM). Operation of the transducer was simulated by ANSYS Mechanical APDL Product Launcher software. The piezoelectric strip was modelled by SOLID5 elements with a matching grid of 1 mm. There are 100 elements taking into account complete piezoelectric *e_ij_*, elastic *c_ij_* and dielectric *ε_ij_* matrices of the PMN-32%PT crystal [43,44,45]. The piezoelectric strip has four nodal degrees of freedom including three displacements in the *x*, *y*, *z* directions and electric voltage *U*. The modelling takes into account free boundary conditions coupling between internal electric and mechanic fields and electrical resistors *R*_1_ and *R*_2_ connected to the electrodes. Please note that in the finite element model the resistor *R*_1_ includes not only separate resistor but also the input electric impedance of the external electric circuit, for example, a preamplifier connected to the top electrode.

It was assumed that the acoustic pressure of the incident wave in air is *p* = 1 Pa. The part of the incident wave pressure *p* is reflected by the active edge of the receiver and the total pressure acting on it is:

(1) $$p_{t}=p+p_{r},$$

where:

(2) $$p_{r}=p\frac{z_{in}-z_{air}}{z_{in}+z_{air}},$$

*p_r_* is the acoustic pressure of reflected wave, *z_in_* is the acoustic input impedance of the transducer, *z_air_* is the acoustic impedance of air. In the case of air-coupled receiver *z_in_* >> *z_air_* and *p_r_* ≈ *p*, e.g., the total pressure on the active surface of the receiver is *p_t_* = 2*p*. Therefore in finite element simulations it was assumed that the total pressure acting on the surface of the receiver is *p_t_* = 2 Pa, what corresponds to the pressure *p* = 1 Pa in the incident wave.

**Figure 2:**
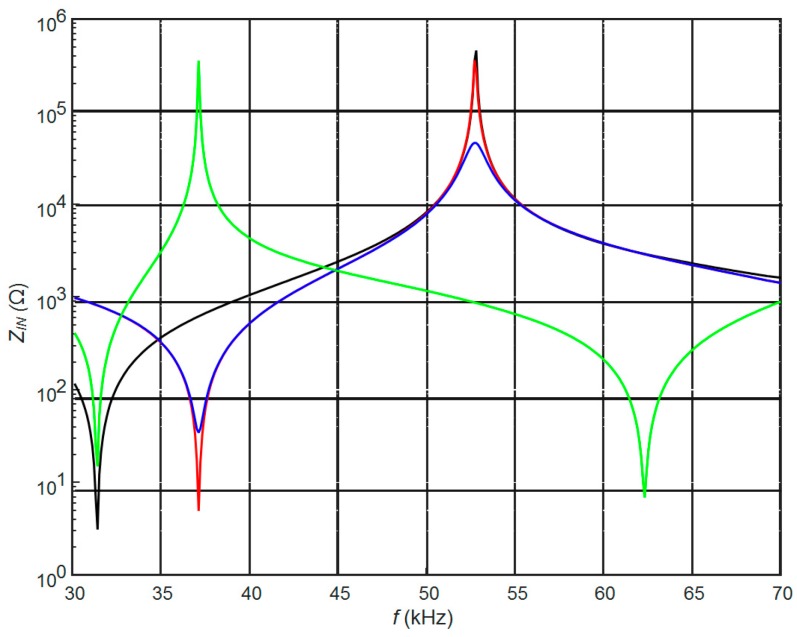
Simulated electric input impedance of the PMN-32%PT 20 × 5 × 1 mm^3^ piezoelement without the matching strip with continuous electrodes (black) and two pairs of rectangular electrodes (90°, model A) with external resistor *R*_2_ connected to the bottom electrodes: *R*_2_ = 1 MΩ (red); *R*_2_ = 50 kΩ (blue); *R*_2_ = 1 Ω (green).

The calculated electric input impedances of the PMN-32%PT 20 × 5 × 1 mm^3^ strip-like piezoelectric element with the continuous and split electrodes are shown in Figure 2. The input electric impedance in the case of split electrodes was calculated on the top electrodes. From the results presented follows that in the case of the continuous electrode the resonance frequency *f_r_* = 31.4 kHz is much lower (Figure 2, black curve) than in the case of two rectangular electrodes of the same dimensions. 

In the case of split electrodes, the resonance frequency depends on the electric load *R*_2_ connected to the bottom electrodes. The biggest changes are observed when the bottom electrodes are almost short-circuited (*R*_2_ = 1 Ω). Then the resonance frequency *f_r_* = 31.4 kHz coincides with the resonance frequency of the piezoelectric element covered by continuous electrodes. Correspondingly reduction of the resistance *R*_2_ from conditions close to the open circuit (*R*_2_ = 1 MΩ) down to the short-circuit conditions (*R*_2_ = 1 Ω) changes the antiresonance frequency from *f_a_* = 52.8 kHz down to *f_a_* = 31.4 kHz. It means that by selecting the resistor *R*_1_ it is possible to control resonance and antiresonance frequencies and the level of damping of the strip-like piezoelectric element.

The frequency response and consequently the pulse response of the ultrasonic receiver essentially depend on a material and dimensions of the acoustic matching strip. The acoustic impedance of the λ/4 length matching element material according to the ideal matching condition [45] should be:

(3) $$Z_{s}=\sqrt{Z_{t}Z_{air}},$$

where *Z_t_* = *ρ_t_c_t_* = 13.52 MRayl is the specific acoustic impedance of the PMN-32%PT strip in the transverse-extension mode in direction 2 (Figure 1), *ρ_t_* = 8100 kg/m^3^—the density, *c_t_* = 1669 m/s is the ultrasound velocity in the transverse-extension mode in direction 2 and *Z*_air_ = 0.445 kRayl is the specific acoustic impedance of air. From Equation (3) follows that the acoustic impedance of the matching strip material should be *Z*_s_ = 0.088 MRayl.

For the acoustic matching strip, we selected rigid polymer foams manufactured by Airex AG (Sins, Switzerland). The acoustic properties of some AIREX type materials are given in Table 1. Ultrasound velocities in AIREX samples were measured by a through-transmission method [46]. Ultrasound attenuation is rather small due to the short length—a quarter wavelength, therefore it has no influence on operation of the transducer and was not measured. The materials possessing the acoustic impedance *Z_s_* closest to this value are AIREX C70.75 and T10.110. From the point of a view of mechanical processing better properties possesses AIREX T10.110, because its rigidity is more suitable for mechanical cutting of matching strips.

Frequency responses of the ultrasonic receivers with two rectangular pairs of electrodes with matching strips made of different AIREX type materials were calculated by FEM. Please note that both resistors *R*_1_ and *R*_2_ for all cases were the same *R*_1_ = *R*_2_ = 50 kΩ. The analyzed cases are presented in Table 2. In this table, the obtained frequency bandwidths and sensitives are also presented.

**Figure 3:**
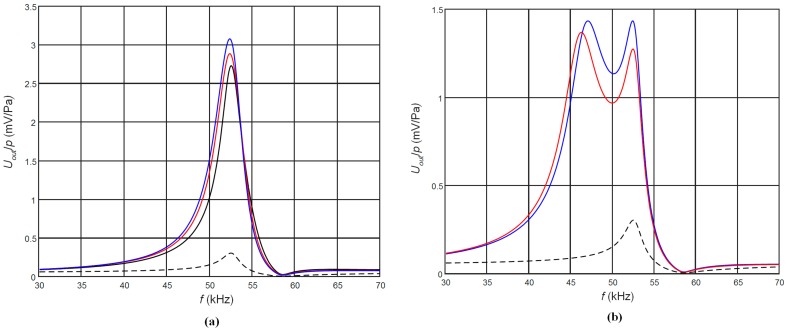
Simulated frequency responses of the PMN-32%PT receivers at the output of the top rectangular electrode (case A, *R*_1_ = 50 kΩ, *R*_2_ = 50 kΩ) with the matching strips the length of which is *l*_1_ = λ/4 at the antiresonance frequency *f_a_* = 53 kHz (**a**) and *f_a_* = 46 kHz (**b**) made of different AIREX type materials: black—C70.75; red—C70.130; blue—T10.100. Black dashed line—without the matching strip.

The corresponding frequency responses are shown in Figure 3. The frequency responses presented in Figure 3a are for the case of the matching strips the length of which is *l*_1_ = λ/4 at the antiresonance frequency *f_a_* = 53 kHz of the piezoelectric strip. Due to different ultrasound velocities in different AIREX foams, those lengths were different (Table 2). The frequency responses were calculated as the electric potential at the output of the top rectangular electrode when the ultrasonic wave incident on the top edge of the receiver is 1 Pa. For comparison, the black dashed line shows the frequency response of the piezoelectric receiver without a matching strip. Please note that in this case the maximum sensitivity is obtained at the antiresonance frequency *f_a_* = 53 kHz. From the results obtained follows that significant improvement up to ten times of the sensitivity is achieved, but the frequency bandwidth is narrow and not suitable for operation in a pulse mode (Table 2). In order to increase the bandwidth we have investigated the cases when the length of the matching strip λ/4 was adjusted to the frequencies other than antiresonance frequency of the piezoelectric strip. The performed modelling showed that in this case in the frequency response another peak appears at the frequency *f_l_* = *c/*4*l*_1_ (Figure 3b). Selecting the proper length of the matching strip *l*_1_ it is possible to control the frequency of this peak and to get a wider bandwidth. The frequency responses when the length of the matching strip *l*_1_ = λ/4 was adjusted to the frequency *f_l_* = 46 kHz are shown in Figure 3b. It is possible to observe significant widening of the bandwidth, which reaches 8 kHz or 16% of the central frequency 50 kHz, but there is still a noticeable non-uniformity of the frequency responses. The ways, in which it is possible to improve the frequency response, we shall explain in the next paragraphs.

In addition, from the point of a view of the sensitivity all listed AIREX materials show a similar performance. Therefore taking into account suitability for a mechanical processing for a further analysis we have selected AIREX T10.110.

As it was pointed out above, the propagation direction of an ultrasonic wave inside the piezoelectric element is orthogonal to the direction of the electric field generated due a piezoelectric effect the internal electric field. It means that acoustic field depends on electric boundary conditions that allow manipulating geometry of the electrodes covering the side surfaces of the piezoelectric element to control frequency and pulse responses of the transducer. 

**Figure 4:**
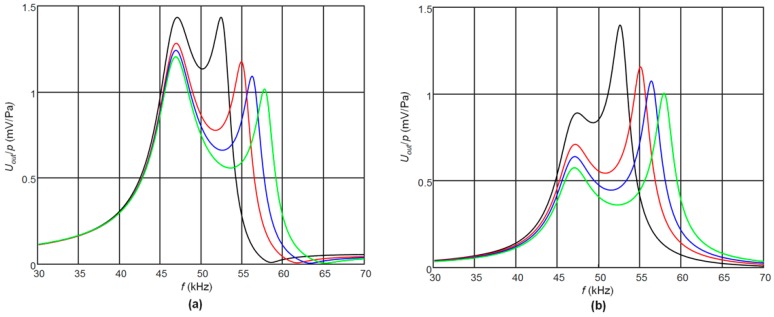
Simulated frequency responses of the PMN-32%PT receivers on the top (**a**) and bottom (**b**) pairs of electrodes with different gaps between electrodes and the matching strip made of AIREX T10.110 the length of which is *l* = 5.7 mm, *R*_1_ = *R*_2_ = 50 kΩ: 1 mm—black color, 2 mm—red, 3 mm—blue, 4 mm—green.

First, we have investigated how the frequency response depends on the width of the gap between two rectangular electrodes. The calculated frequency responses of the PMN-32%PT receivers with gaps of different width between the two pairs of electrodes are presented in Figure 4a,b. In Figure 4a are shown frequency responses on the top pair of electrodes (closest to the matching strip), in Figure 4b—on the bottom electrodes. It is possible to see that the frequency responses observed on two pairs of electrodes have a similar character except that the second peak in the frequency responses is changing in opposite manner—on the top pair of electrodes it is reducing with the increasing gap, but on the bottom pair of electrodes it is increasing. From the results obtained follows that with increasing the gap the potential frequency bandwidth also increases, however non-uniformity of the responses is increasing significantly.

**Figure 5:**
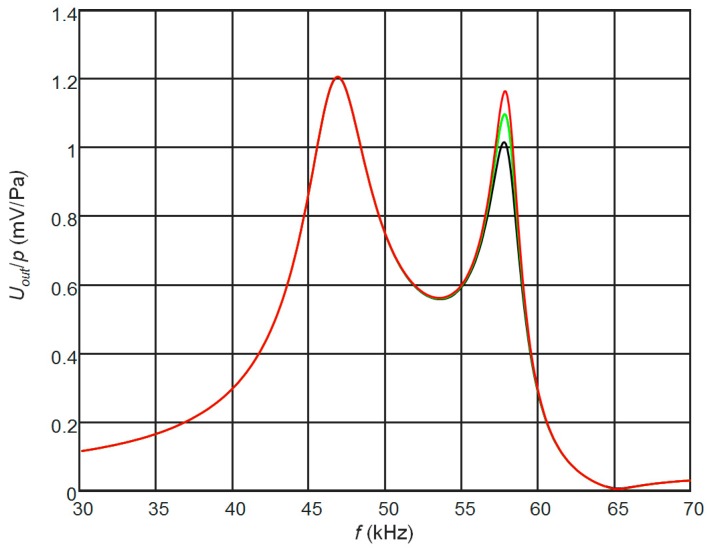
Simulated influence of the resistor *R*_2_ connected to the bottom electrode on the frequency responses of the PMN-32%PT receivers on the top electrode (the gap between electrodes *l*_3_ = 4 mm, length of the matching strip AIREX T10.110 λ/4 (5.7 mm), *R*_1_ = 50 kΩ): *R*_2_ = 50 kΩ—black; *R*_2_ = 60 kΩ—green, *R*_2_ = 70 kΩ—red.

From simulations performed by FEM follows that a frequency response of the receiver also depends on the electrical resistors *R*_1_ and *R*_2_ connected to the top and bottom electrodes. It is due to the dissipation of the electrical energy of the converted acoustic signal by the resistors *R*_1_ and *R*_2_. The resistor *R*_2_ connected to the bottom electrodes creates an additional damping of the piezoelectric strip, which may be called an electromechanical damping. It means that changing the level of the electromechanical damping by means of the resistor *R*_2_ we can expect to flatten the frequency response what would be beneficial for operation of the receiver in a transient mode. The frequency responses of the receiver with different resistors *R*_2_ connected to the bottom electrode are shown in Figure 5. The resistor *R*_1_ which takes into account the input impedance of the preamplifier in all cases was the same *R*_1_ = 50 kΩ. We can see that the amplitude of the second peak in the frequency response at 57 kHz really depends on the resistor *R*_2_, however it is still impossible to flatten sufficiently those responses only by selection of the electric load.

**Figure 6:**
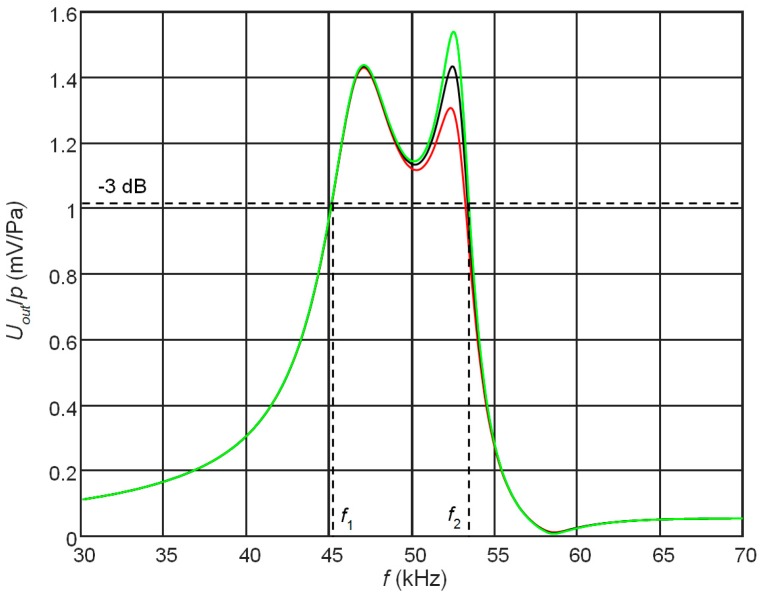
Simulated frequency responses of the PMN-32%PT receivers on the top pair of electrodes at different electromechanical damping levels (length of the matching strip AIREX T10.110, λ/4 (*l* = 5.7 mm), *R*_1_ = 50 kΩ): red—*R*_2_ = 40 kΩ, black—*R*_2_ = 50 kΩ, green—*R*_2_ = 60 kΩ; *f*_1_ = 45.1 kHz, *f*_2_ = 53.1 kHz, the bandwidth is ∆*f* = 8 kHz.

Therefore, the best solution is to keep the gap narrow, for example 1 mm, and to modify the frequency response by selecting the electric resistances of the resistors *R*_1_ and *R*_2_ connected to the both electrodes. The simulated frequency responses in this case are presented in Figure 6.

The shown frequency responses were obtained at different values of the resistor *R*_2_ connected to the bottom electrodes (Figure 1). Please note that higher values of the resistor *R*_2_ give lower damping and the second peak amplitude increases. From the results presented follows that controlling level of the electromechanical damping allows to achieve a quite wide frequency bandwidth ∆*f* = 8 kHz. The further improvement of the frequency response and the bandwidth is possible by changing geometry of the electrodes. This is discussed in the next paragraph.

## 3. Piezoelectric Receiver with Two Pairs of Non-Rectangular Electrodes

The PMN-32%PT receiver with two non-rectangular electrodes and with the λ/4 matching strip is shown in Figure 1c. The gap between electrodes may be oriented at different angles. We have performed FEM simulations in the range of angles from 35° to 65° and have found that in all directions of the gap additional modes in the plane of the piezoelectric strip are excited, which cause strong artefacts in the frequency response. The highest sensitivity and widest bandwidth is obtained at the 45°. Therefore, we have analyzed the case with the gap oriented at 45°. The electric input impedance, sensitivity and frequency responses of the receiver depend on dimensions of both the top and bottom pairs of electrodes. For estimation of this influence, we have analyzed three cases B_1_, B_2_ and B_3_ (Table 3, Figure 1b).

**Figure 7:**
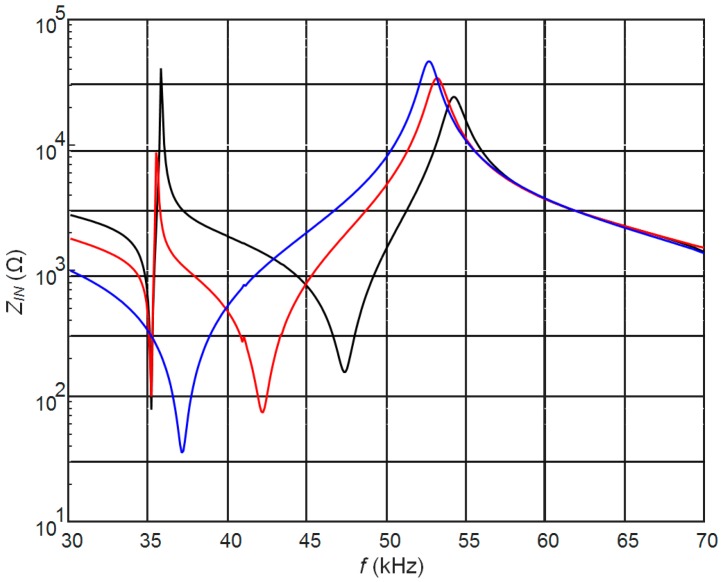
Simulated electric input impedance on the top electrodes of the PMN-32%PT 20 × 5 × 1 mm piezoelement without the matching strip, with two pairs of non-rectangular electrodes of different dimensions and 45° gap, *R*_2_ = 50 kΩ: black—case B_1_; red—case B_2_, blue—case B_3_.

The width of the gap *l*_3_ between electrodes along *y* axis in all cases was the same *l*_3_ = 1 mm (Figure 1). The calculated electric input impedance of the PMN-32%PT piezoelement without the matching strip is presented in Figure 7. Comparison of this impedance with the electric impedance of the transducer with continuous and two pairs of rectangular electrodes (Figure 2) clearly shows significant differences. The dimensions of the electrodes influence antiresonance frequencies, which for presented cases are the following: case B_1_—*f_a_*_1_ = 54.2 kHz; case B_2_—*f_a_*_2_ = 53.2 kHz; case B_3_—*f_a_*_3_ = 52.7 kHz.

**Figure 8:**
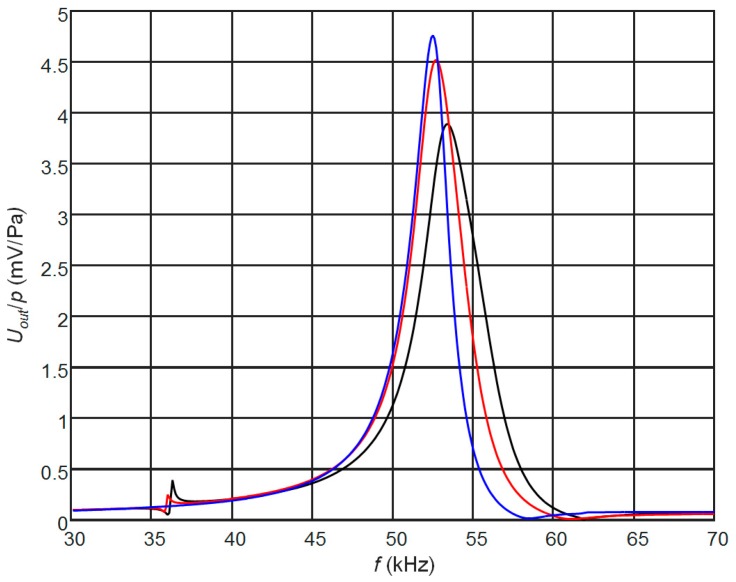
Simulated influence of electrodes dimensions on the frequency response observed on the top electrodes (AIREX T10.110, *R*_1_ = 1 MΩ, *R*_2_ = 50 kΩ): black color—case B_1_ (λ/4 = 4.9 mm); red—case B_2_ (λ/4 = 5.0 mm), blue—case B_3_ (λ/4 = 5.1 mm).

The frequency responses for those cases when the length of the matching strip λ/4 was adjusted to the different antiresonance frequencies *f_a_*_1_, *f_a_*_2_ and *f_a_*_3_ are presented in Figure 8. Those results are quite similar to the modelling results in the case of two rectangular electrodes of equal area (Figure 3a) except that an additional peak in the frequency responses close to the frequency of 35.5 kHz is observed. By means of the finite element modelling, we have determined that this peak is due to the additional vibration mode excited in the plane of the piezoelectric strip by a sharper corner of the non-rectangular electrodes. The biggest displacements are in the *x* direction. Amplitude of this mode is reducing with the increasing angle of the electrodes cusp. In the case of the 45° gap the amplitude of this artefact depends on dimensions of the top and bottom electrodes and it completely disappears when the areas of the electrodes are equal, e.g., case B_3_. In this case the highest sensitivity is obtained, but the observed bandwidth is rather narrow −1.9 kHz and is not suitable for operation in a pulse mode.

**Figure 9:**
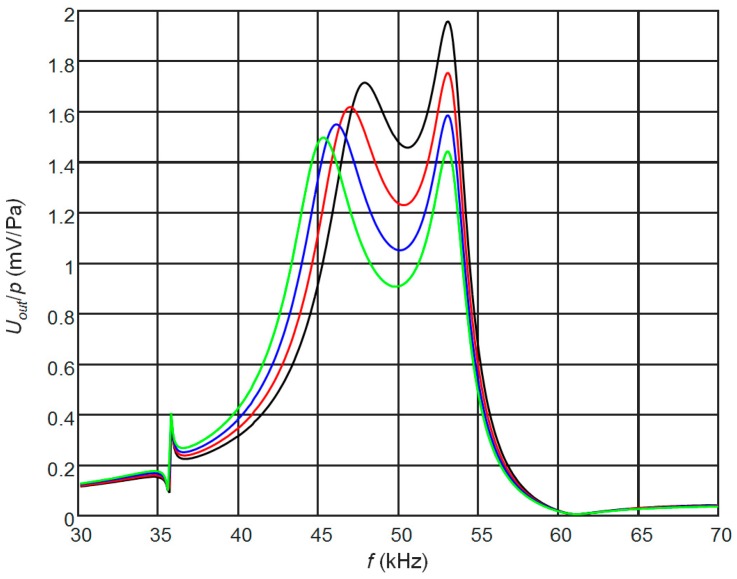
Simulated frequency responses of the PMN-32%PT receivers at the output of the top non-rectangular electrode (case B_2_, 45° gap) with the λ/4 length matching strips (AIREX T10.110), *R*_1_ = *R*_2_ = 50 kΩ, adjusted to different frequencies: black—*f_s_* = 47.6 kHz, *l*_1_ = 5.6 mm; red—*f_s_* = 46.7 kHz, *l*_1_ = 5.7 mm; blue—*f_s_* = 45.9 kHz, *l*_1_ = 5.8 mm, green—*f_s_* = 45.2 kHz, *l*_1_ = 5.9 mm.

The frequency bandwidth may be improved adjusting the length of the matching strip λ/4 to the frequencies lower than the antiresonance frequency *f_a_*. In those cases, the length of matching strips is different (Figure 9) and the potential bandwidth may be significantly wider, however they are non-uniform.

**Figure 10:**
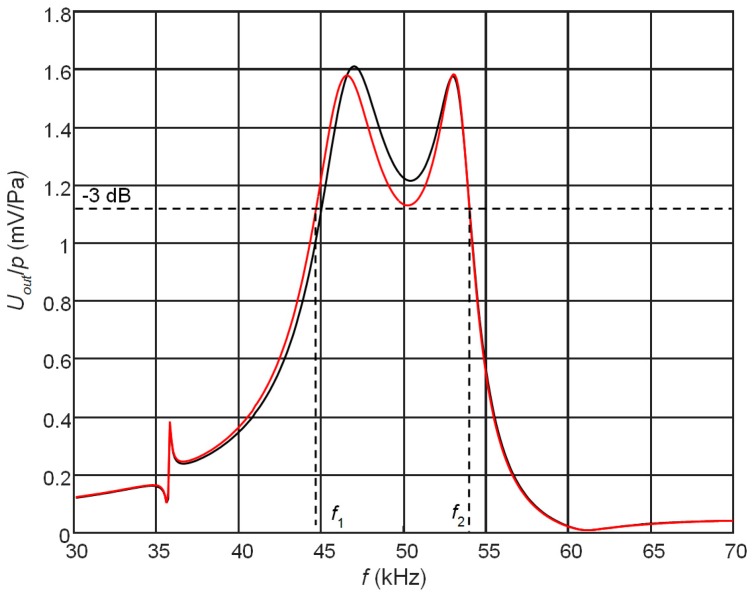
Simulated frequency responses of the PMN-32%PT receivers at the output of the top non-rectangular electrodes (case B_2_, 45° gap) with the AIREX T10.110 (*c* = 1066 m/s) λ/4 matching strips of a different length: black—*l*_1_ = 5.70 mm, *f_s_* = 46.7 kHz (*R*_1_ = 50 kΩ, *R*_2_ = 40 kΩ); red—*l*_1_ = 5.75 mm, *f_a_* = 46.3 kHz (*R*_1_ = 50 kΩ, *R*_2_ = 45 kΩ), *f*_1_ = 44.7 kHz, *f*_2_ = 53.9 kHz, ∆*f* = 9.2 kHz.

Uniformity of the frequency responses can be improved selecting the proper length of the matching strip *l*_1_ and adjusting electromechanical damping by means of the resistor *R*_2_. In this case, a trade-off between the sensitivity and bandwidth of the receiver can be obtained (Figure 10). The results presented in Figure 10 show that in this case a quite wide bandwidth ∆*f* = 9.2 kHz or 18.4% with respect to the central frequency 50 kHz is obtained. For air-coupled ultrasonic transducer it is good result.

**Figure 11:**
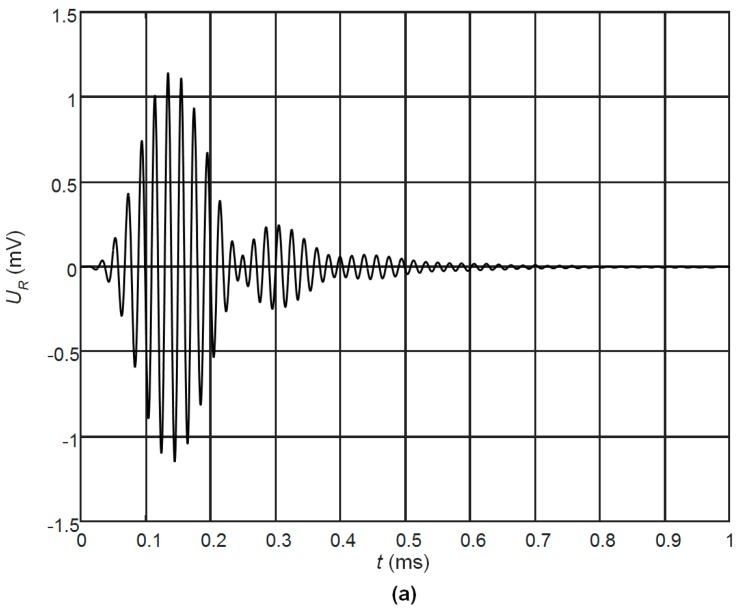

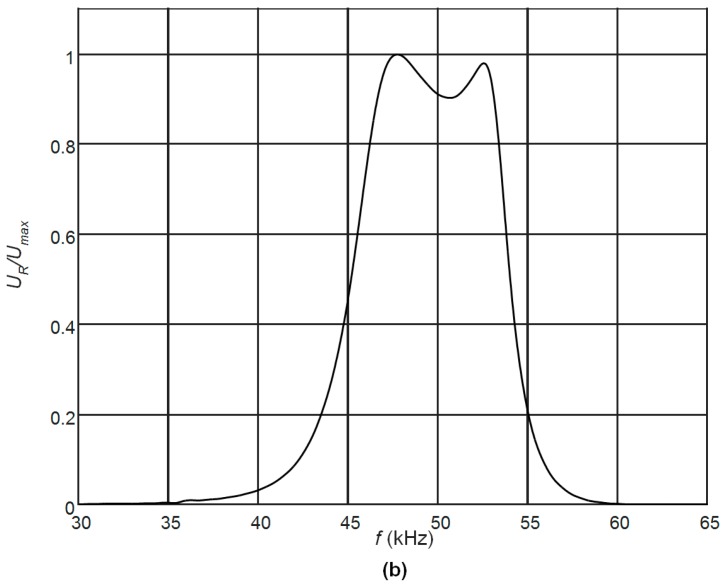
Simulated waveform (**a**) and the spectrum (**b**) of the electric potential on the top electrodes (*R*_1_ = 50 kΩ, *R*_2_ = 40 kΩ, number of periods *n* = 5, excitation frequency *f* = 50.0 kHz, λ/4 matching strip-AIREX T10.110 (*l* = 5.70 mm).

The pulse response of the receiver with the optimal matching strip *l* = 5.75 mm and two non-rectangular pairs of electrodes to the excitation by the 5 periods of 50 kHz ultrasonic pulse with the amplitude of 1 Pa is presented in Figure 11a. The spectrum of the received signal is shown in Figure 11b. Please note, that the width of this spectrum due to a band limited spectrum of the excitation pulse is ∆*f* = 7.5 kHz. The presented results demonstrate a good performance of the developed air-coupled receiver in a pulse mode also.

## 4. Experimental Investigation

**Figure 12:**
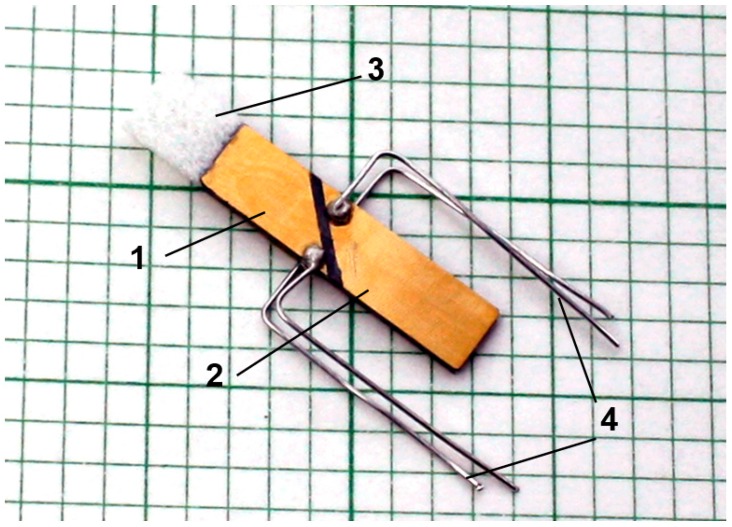
The PMN-32%PT crystal receiver element (case B_2_, 45° gap) with non-rectangular top (1) and bottom (2) electrodes, λ/4 length matching strip of the AIREX T10 110 (3) and electrical wiring (4).

The experimental investigation of the proposed air- coupled ultrasonic receiver (Figure 1c) was performed using PMN-32%PT crystals plates with <011> cut and [001] poling direction from the H.C. Materials Corporation (HCMC, Bolingbrook, IL, USA). The strips-like piezoelectric elements of necessary dimensions 20 × 5 × 1 mm^3^ were cut of the 20 × 20 × 1 mm^3^ poled PMN-32%PT crystal and were coated by gold electrodes. The necessary non-rectangular shapes of electrodes were obtained by the “aqua regia”, e.g., nitrohydrochloric acid etching technique [47] of the 45° gaps whole electrodes. The piezoelectric PMN-32%PT receiver element prepared for experimental investigation is shown in Figure 12.

The two pairs of electrodes were connected to electrical circuits by electrical semi-rigid wires connected to the electrodes using the 3022 type e-solder from Von Roll Isola (Shenectady, NY, USA, (Figure 12). Those wires also serve as a support of the piezoelectric elements in the experimental transducer set. The matching λ/4 strip made of AIREX T10.110 was glued to the PMN-32%PT element using cyanoacrylate type glue (FixPoint, Braunschweig, Germany). During investigation, the piezoelectric strip and wiring with electrical connectors were placed into a housing, which shields them from electromagnetic and acoustical fields. Only the front edge of the matching layer contacted with air environment. 

**Figure 13:**
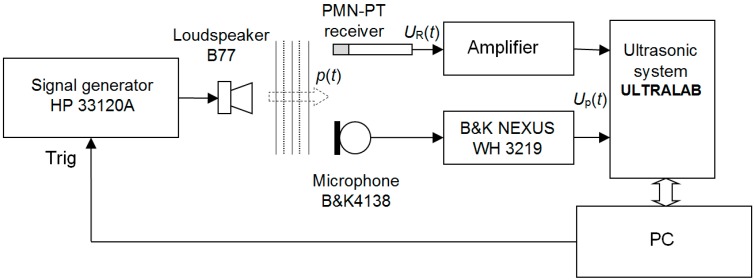
Schematic diagram of the experimental set-up for investigation of the PMN-PT receiver.

The purpose of the experiments was to check how mathematical modelling is close to the experimental results and to estimate performance of the receiver. The experiments were performed with the set-up presented in Figure 13. 

As a source of ultrasonic waves the high frequency tweeter of the multimedia speaker B77 from the Microlab (Shenzhen, China) was used. The HP 33120A type function/arbitrary waveform generator (HP 33120A, Hewlett-Packard, Palo Alto, CA, USA) excites this loudspeaker by the voltage burst of a selected frequency and shape and generates an acoustic pressure burst *p(t)*. In order to eliminate the influence of the frequency response of the loudspeaker B77 on measurement results reception of the radiated ultrasonic wave is performed by two measurement channels. One channel used as a reference channel consists of the B&K4138 type microphone and B&K NEXUS WH 3219 type amplifier (Brüel & Kjær, Naerum, Denmark) and enables to perform absolute measurements of the acoustic pressure in the frequency band up to 140 kHz. The investigated PMN-32%PT receiver is connected to the second channel. The signals from both channels were stored, processed and analysed by the ultrasonic measurement system ULTRALAB (Ultrasound Institute, Kaunas University of Technology, Kaunas, Lithuania). 

**Figure 14:**
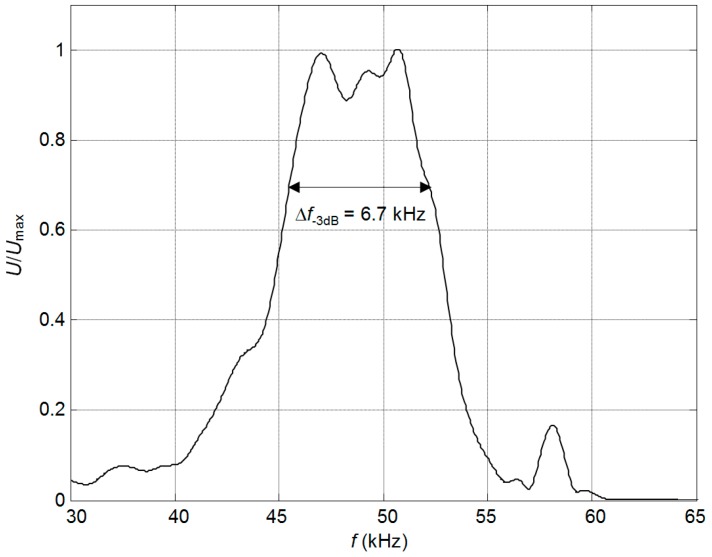
Experimentally determined normalized frequency response of the PMN-32%PT strip-like receiver with two non-rectangular electrodes (gap between electrodes at 45° angle, case B_2_).

The measured frequency response of the PMN-32%PT strip-like receiver with a matching strip made of AIREX T10.110 with the length *l* = 5.9 mm and two non-rectangular electrodes (Figure 1c) is presented in Figure 14. The measured bandwidth of the receiver at −3 dB level is ∆*f* = 6.7 kHz or (∆*f*/*f*_0_) 100% = 14% with respect to the central frequency *f*_0_ = 49 kHz. It is narrower than the obtained in the simulation bandwidth ∆*f* = 7.5 kHz (Figure 11b), because influence of the glue between the piezoelectric element and the matching strip in the simulation was not taken into account. The obtained relative frequency bandwidth is wider than of the ultrasonic transducers used in air-coupled NDT [20]. For example, the frequency bandwidth of the air-coupled transducers AirTech50 with the central frequency 50 kHz and used for NDT of aerospace structures is only ∆*f* = 5 kHz or 10% of the central frequency [20].

**Figure 15:**
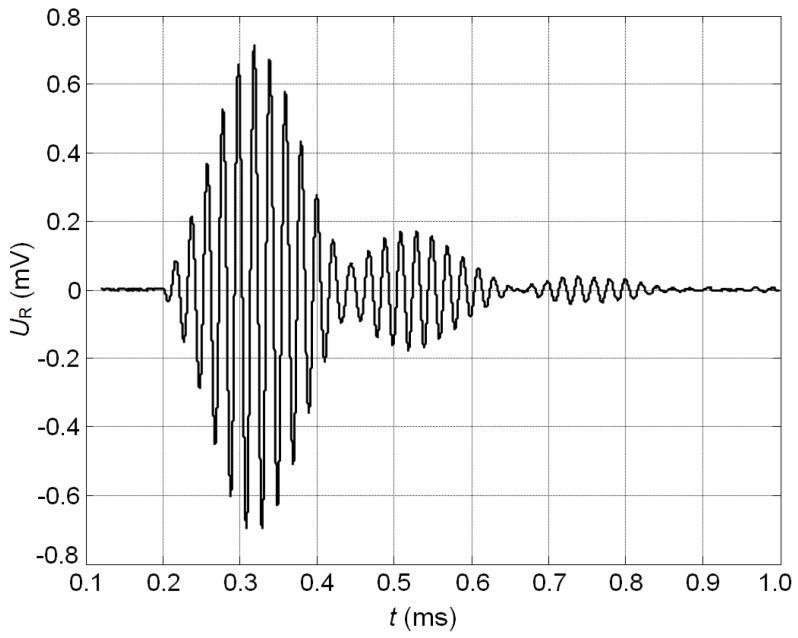
Waveform of the ultrasonic signal at the output of the receiver with two non-rectangular electrodes (gap between electrodes at 45°, case B), when the acoustic excitation signal was the five periods sine burst with the acoustic pressure amplitude of *p* = 1.0 Pa.

The ultrasonic pulse with a rectangular envelope radiated by the loudspeaker B77 duration of which was five periods of 51 kHz was picked up by the developed PMN-32%PT receiver. Figure 15 shows the waveform of the electric signal at the output of the receiver. The obtained results demonstrate a good performance of the air-coupled ultrasonic receiver both in harmonic and transient modes. 

Comparison of the simulation and experiment’s results of the PMN-32%PT transducer with the matching strip made of the AIREX T10.110 are presented in Table 4. 

Presented comparison of the experimental results with the modelling results indicates a rather good correspondence (Figure 11a and Figure 15).

## 5. Conclusions

For NDT of composite structures various modes of guided waves are already used. For their air-coupled excitation and reception multi-element arrays may be employed [41,42]. In a frequency range below 100 kHz, arrays with strip-like piezoelectric elements, vibrating in the transverse-extension mode may be used. Such array allows exciting anti-symmetric modes with velocities close or even lower than ultrasound velocity in air [41]. Operation of such transducers and arrays in a transmitting mode was discussed in detail in [42]. However, operation of ultrasonic transducers with piezoelectric elements possessing a high electromechanical coupling in transmitting and receiving modes is rather different. Therefore, for transmission and reception of ultrasonic signals separate piezoelectric elements with different dimensions must be used. Performance of piezoelectric receivers made of the PMN-32%PT strip-like elements vibrating in a transverse-extension mode may be efficiently controlled by selecting geometry of the electrodes covering side surfaces of the piezoelectric element. It is equivalent to introduction of electromechanical damping which does not require any additional backing element. For this purpose, the continuous electrodes are divided into two pairs of electrodes. The one pair is used to pick up the electric signal, another one is exploited for electromechanical damping. The level of damping may be controlled by the electric resistor connected to the damping pair of electrodes. Two types of electrodes may be used-rectangular or non-rectangular with a gap between them directed at some angle, usually 45°. The better performance is observed with non-rectangular electrodes. The performed FEM simulations showed that at other directions of the gap additional modes in the plane of the piezoelectric strip are excited, which causes strong artefacts in the frequency response, therefore the angle was selected 45°. Performance of the ultrasonic receiver may be significantly enhanced by acoustic matching strip-like elements bonded to the tip of the PMN-32%PT crystal. It was proposed to use for this purpose AIREX T10.110 rigid polymer foam the acoustic impedance of which is close to the optimal value necessary for matching with air. Usually the length of the matching element is selected λ/4 at the operation frequency. However, it was found that in order to get a wide bandwidth the length of the matching strip should be selected not a quarter wavelength λ/4 at the antiresonance frequency *f_a_* but at lower frequency. In such a way it was possible to achieve the frequency bandwidth at −3 dB level (14–18)% with respect to the central frequency *f*_0_ = 50 kHz. It is necessary to point out that by reducing the length of the piezoelectric strips is possible to achieve higher operation frequencies up to 150 kHz. The pulse response is rather short (5 to 7 periods) and is suitable for many air-coupled NDT techniques. The developed and investigated ultrasonic receivers are intended to use in multi element linear arrays in a pitch-catch mode together with the developed transmitting array [44].

## Figures and Tables

**Table 1:** Properties of polymers used in matching elements.

Properties	AIREX C70.75	AIREX C70.130	AIREX C70.200	AIREX T10.110
Density *ρ* (kg/m^3^)	80	130	200	110
Ultrasound velocity *c* (m/s)	1140	1143	1183	1066
Acoustic impedance *Z* (MRayl)	0.091	0.149	0.236	0.117

**Table 2:** Properties of ultrasonic receivers with matching strips made of different AIREX type materials.

Material of Matching Strip	Antiresonance Frequency *f_a_* = 53 kHz			Frequency *f_a_* = 46 kHz		
	Length *l*_1_ = λ/4 (mm)	Bandwidth (kHz)	Sensitivity (mV/Pa)	Length *l*_1_ = λ/4 (mm)	Bandwidth (kHz)	Sensitivity (mV/Pa)
C70.75 (black)	5.4	2.1	2.73	-	-	-
C70.130 (red)	5.4	2.6	2.89	6.2	8.3	1.44
T10.110 (blue)	5.1	2.7	3.08	5.7	8.2	1.47

**Table 3:** Piezoelectric receiver with two pairs of non-rectangular electrodes.

Dimensions	Case B_1_	Case B_2_	Case B_3_
*l*_2_ (mm)	7.5	9.5	12
*l*_4_ (mm)	11.5	9.5	7
The area of the top electrode *S*_1_ (mm^2^)	25	35	46.5
The area of the bottom electrode *S*_2_ (mm^2^)	70	60	46.5

**Table 4:** Comparison of the simulation and experiment results.

Properties	Simulation	Experiment
Bandwidth, kHz	7.5	6.7
Sensitivity, mV/Pa	1.1	0.77
